# Investigation of the Frequency of Coronary Artery Anomalies in MDCT Coronary Angiography and Comparison of Atherosclerotic Involvement between Anomaly Types

**DOI:** 10.3390/tomography8030135

**Published:** 2022-06-20

**Authors:** Tuna Şahin, Mehtap Ilgar

**Affiliations:** 1 Department of Radiology, Faculty of Medicine, Adnan Menderes University, 09100 Aydın, Turkey; 2 Department of Radiology, Malatya Training and Research Hospital, 44330 Malatya, Turkey; mehtapilgar@gmail.com

**Keywords:** coronary artery anomaly, MDCT angiography, coronary atherosclerosis

## Abstract

Coronary artery anomalies (CAAs) are rare anatomical variations characterized by abnormal origin, course, or termination of the coronary arteries. This study aims to identify incidental CAAs in patients who underwent multidetector computed tomography coronary angiography (MDCTCA) to determine their incidence and to evaluate whether there is a difference between CAA types in terms of coronary atherosclerotic involvement. For this purpose, patients who underwent MDCTCA between December 2018 and January 2022 were retrospectively assessed. Of the 5200 MDCTCAs analyzed, CAAs were detected in 136 patients (2.61%). Of these 136 patients, 37 (27.2%) patients had an origin anomaly, 97 (71.3%) had a course anomaly, and 2 (1.5%) had a termination anomaly. There was no statistically significant difference between CAA types in terms of atherosclerotic involvement (*p* = 0.220). However, atherosclerotic involvement was high in vessels with anomalies when normal vessels with and without anomalies were compared (*p* = 0.005). Accurate detection of CAAs is vital for endovascular treatment or surgical intervention. MDCTCA is helpful both in the diagnosis of CAA and in the early detection and development of prevention strategies for coronary atherosclerosis.

## 1. Introduction

Coronary artery anomalies (CAAs) are congenital disorders with different clinical presentations and might be associated with congenital heart diseases. Most anomalies are detected accidentally by multidetector computed tomography coronary angiography (MDCTCA) or conventional coronary angiography (CCA) [1]. Their incidence has been recorded as 0.6% to 5.64% in angiographic series and 0.3% in autopsy data because of geographical variation [2]. The increased frequency of CAAs in adults has been attributed to the extensive use of coronary imaging for the evaluation of ischemic heart disease. Although it is mostly asymptomatic, arrhythmia, syncope, myocardial infarction, and sudden cardiac death can be seen, especially in young individuals [3]. It has also been reported that life-threatening ventricular arrhythmias, electrical disturbances, and sudden cardiac death due to thoracic aortic dissection can be seen during physical exercise, especially in young adult athletes with adolescent and hypertrophic cardiomyopathy [4,5,6,7,8].

Since CAAs are rare, current data on their clinical profile and management are insufficient in the literature. However, they can be better identified, especially with the introduction of MDCTCA into clinical use [9]. Many terminologies and classifications have been proposed for the definition, terminology, and classification of CAAs [2]. Greenberg et al. classified CAAs into three groups (shown in Table 1) as anomalies of termination, course, and origin [10].

It is essential to be able to accurately identify coronary anatomy and coronary artery stenosis to avoid complications such as incorrect ligation or transection during angioplasty or bypass [2]. There are studies in the literature investigating atherosclerotic involvement in abnormal coronary arteries [11,12]. The leading cause of chest pain is atherosclerotic disease. Moreover, myocardial ischemia can be seen in the absence of atherosclerotic disease in cases such as inter-arterial course of the coronary artery, myocardial bridging, vasculitis, coronary artery dissection, and abnormal termination of the left coronary artery from the fistula and pulmonary artery. The MDCTCA is a non-invasive diagnostic technique for assessing coronary arteries in people who are at low to moderate disease possibility of obstructive coronary artery disease. It is also useful for demonstrating non-atherosclerotic reasons for obstruction, especially abnormalities of coronary artery termination, course, and origin, as well as evaluating their relationship with surrounding structures. Today, the CAD-RADS scoring system is used in MDCTCA reports to ensure consistency, communicate effectively with clinicians, and provide reliable data for scientific studies and teaching. There are six levels in the CAD-RADS scoring system, ranging from 0 (no plaque) to 5 (1 total occlusion minimum). The maximum degree of stenosis available determines the group. Additional data about plaque sensitivity, bypass grafts, stents, and non-diagnostic picture quality are provided by four modifiers [13].

Both coronary anatomy and coronary artery stenosis are identified non-invasively with MDCTCA. The present study was carried out to determine incidental CAAs in patients who underwent MDCTCA, to determine their incidence, and to evaluate whether there is a difference between CAA types in terms of coronary atherosclerotic involvement.

## 2. Materials and Methods

Our institutional review board authorized this retrospective study methodology, and the research was carried out in accordance with the Declaration of Helsinki. This single-center study is based on coronary MDCT angiography data from the Adnan Menderes University School of Medicine, Radiology Department database. MDCTCA examinations performed between December 2018 and January 2022 were retrospectively reviewed. Patients with inadequate diagnostic image quality and examinations conducted for purposes other than coronary artery evaluation (congenital heart disease, evaluation of cardiac/pulmonary veins, cardiac masses, investigation, surgical planning, valve evaluation, and calcium scoring) were excluded from the research. CAAs were observed in 136 of the 5200 MDCTCA examinations that met the study criteria. All CAA patients’ hospital records were obtained from the institution’s database and 136 patients with CAAs were included in the study. Demographic information and cardiovascular risk factors of the patients were obtained from the hospital data-processing system. MDCTCA images were evaluated at the workstation by a radiologist with 12 years of experienced in cardiac imaging. Anomalies were grouped as anomalies of termination, course, and origin according to the categorization system suggested by Greenberg et al. [10]. All coronary arteries with and without anomalies in each group were analyzed and categorized by the CAD-RADS classification in terms of atherosclerotic involvement, degree of coronary obstruction, type of plaque, and presence and patency of bypass grafts and stents.

### 2.1. MDCTCA Scanning Protocol

A 160-slice computed tomography, 128-detector equipment was used for all MDCTCA tests (Aquilion Prime, Toshiba Medical Systems, Otawara, Japan). First, 70–100 mL of iohexol 350 mg/mL iodinated contrast material was injected into the left antecubital vein at a rate of 4 mL/s with an automated injection device. Following the beginning of contrast agent infusion, an MDCT scan was performed using the bolus tracking approach from the apex of the heart to the baseline. All MDCTCA studies were performed in the craniocaudal supine position within a single inhalation period. Examinations were performed using prospective or retrospective modulation accompanied by electrocardiography (ECG). In the retrospective modulation, all phases from 0% to 90% were assessed by reconstructing the R-R interval with 10% intervals.

### 2.2. MDCTCA Imaging Protocol for Coronary Artery Disease

Section thickness: 0.5 mm, rotation time: 400 ms, 300–400 mAs, 100 kVp, and section spacing: 0.25 mm. Axial MDCT sections were transferred to the workstation and examined using 3D volume rendering and maximum-intensity projection (MIP) as well as 2D multiplanar reconstructions (MPRs). A special cardiac analysis program (Terarecon-Aquarius Workstation Intuition Edition v.4.4.7.1021.7056) was used for vessel segmentation.

### 2.3. Statistical Analyses

SPSS v.22 (SPSS Inc., Chicago, IL, USA) was utilized for the statistical analysis. Mean and standard deviation values of continuous variables, numbers, and percentages of categorical data were calculated. According to the anomaly type, the participants were separated into three groups: origin, course, and termination anomalies. Patients with origin anomalies and course anomalies were compared using the chi-square test. Since the number of patients with termination anomalies was low (2 patients), they were not included in the chi-square analysis. The statistical significance level for all findings was *p* < 0.05.

## 3. Results

CAAs were detected in 136 of the 5200 patients who underwent MDCTCA (overall incidence of 2.61%). Of the patients with CAAs, 78 (57.4%) were male and 58 (42.6%) were female. The patients were aged between 24 and 85 years with a mean age of 57.6 ± 11.9 years. There was an origin anomaly in 37 patients (27.2%), course anomaly in 97 patients (71.3%), and termination anomaly in 2 (1.5%) patients. Anomaly types and percentage distributions are presented in Table 2.

All CAA patients with coronary artery disease risk factors were asymptomatic and none of them participated in exercise practice.

Regarding the risk factors for coronary artery disease, hypertension was present in 64 participants (47.1%), diabetes mellitus in 25 participants (18.4%), hypercholesterolemia (total cholesterol > 200 mg/dl) in 60, smoking in 48 (35.3%), and family history in 58 (42.6%). There was no significant difference between the anomaly types regarding the presence of these factors (Table 3).

**Figure 1:**
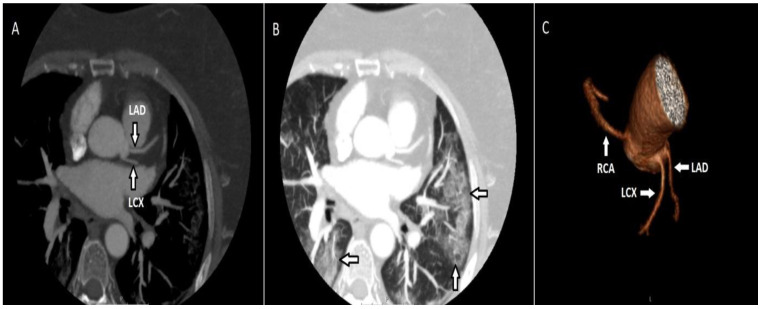
Maximum-intensity projection (MIP) of the axial CT image (**A**) demonstrates the LCX and LAD emerging from the left coronary sinus with separate ostia. The same patient had ground glass infiltrates consistent with COVID-19 (**B**). On the volume-rendered (VR) image (**C**), the LAD and LCX emerge from the left coronary sinus, along with the RCA emerging from the right coronary sinus.

There were 3 patients (2.2%) with high outlet anomalies among the participants with origin anomalies. In these participants, between the pulmonary artery and the aorta, the right coronary artery (RCA) had an inter-arterial course after a high outlet. There were 12 patients (8.8%) with multiple ostium anomalies. In these patients in whom the left main coronary artery (LMCA) was not observed, the left circumflex (LCX) artery and the left anterior descending artery (LAD) emerged from the left sinus with separate ostia. In addition, there were infiltrations compatible with COVID-19 in the thorax sections that entered the examination area, but the patient was asymptomatic. The diagnosis of COVID-19 was confirmed by PCR test (Figure 1).

**Figure 2:**
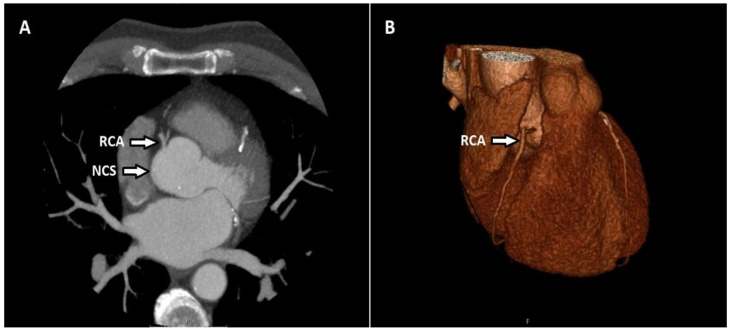
Axial CT maximum-intensity projection (MIP) (**A**) and volume-rendered (VR) (**B**) image shows the RCA originating in the noncoronary sinus (NCS).

There were 2 patients (1.47%) with a singular coronary artery anomaly with the artery starting in the right sinus. There was 1 patient (0.73%) in whom the coronary artery originated from the pulmonary artery. In this patient, the RCA originated from the pulmonary trunk (ARCAPA syndrome). The coronary artery originates from the contralateral or noncoronary sinus in 19 patients (13.9%). Fourteen of these patients had an RCA originating in the left coronary sinus and all had an inter-arterial course after termination. Two of them had LCX originating from the sinus of right coronary and both had a retro-aortic course after their termination. One had an LMCA originating from the right coronary sinus. Two participants had an RCA originating in the noncoronary sinus (Figure 2).

**Figure 3:**
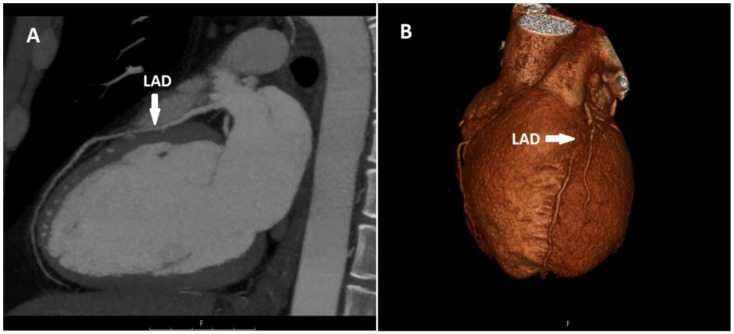
Sagittal CT maximum-intensity projection (MIP) (**A**) and volume-rendered (VR) (**B**) image shows myocardial bridging in the proximal segment of the LAD.

There were 57 patients (41.9%) with myocardial bridging among the patients with course anomalies. Myocardial bridging was present in only the LAD in 54 of these patients, and in both the LAD and LCX in 3 of them (Figure 3).

**Figure 4:**
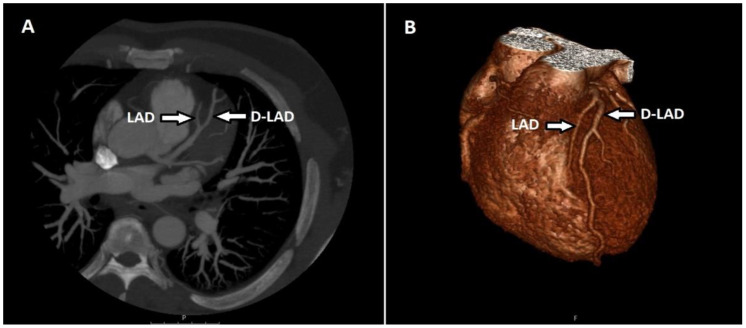
Maximum-intensity projection (MIP) of axial CT (**A**) and volume-rendered (VR) (**B**) image shows the LAD terminating above the atrioventricular groove and the duplicated LAD (D-LAD) extending distally along the groove.

Of the two patients with myocardial bridging only in the LAD, one had a fusiform aneurysm in the LAD and the other in the LCX. There were 40 patients (29.4%) with artery duplications, and all were LAD duplications (Figure 4).

**Figure 5:**
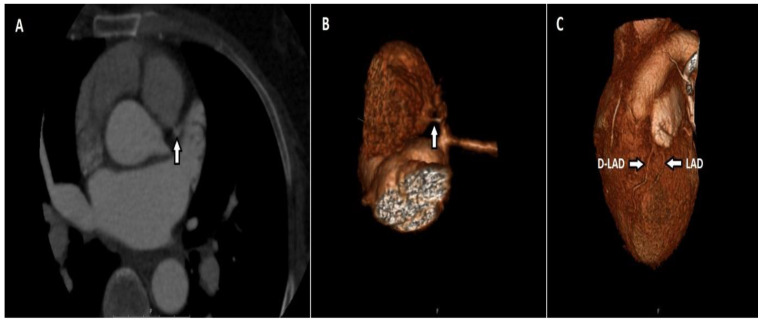
Axial CT maximum-intensity projection (MIP) (**A**) and volume-rendered (VR) (**B**) image shows a thin fistula line between the LAD and the main pulmonary artery. A duplicated LAD (D-LAD) with an LAD is seen in the same patient (**C**).

There were two patients (1.5%) with coronary artery fistula among those with termination anomalies. The first patient had a fistula and accompanying PDA aneurysm between the left atrium and posterior descending artery (PDA). In the other patient, there was a fistula between the pulmonary artery and the LAD, and there was concomitant LAD duplication (Figure 5). No patient had a non-cardiac termination anomaly.

The CAD-RADS scoring system and the degree of atherosclerotic disease as well as the number of affected vessels in patients with origin, course, and termination anomalies are shown in Table 4. Significant coronary artery disease (CAD-RADS 3, 4, and 5) was present in 8 patients (21.6%) with origin anomalies and in 12 patients (12.4%) with course anomalies. None of the patients with termination anomalies had significant coronary artery disease. There was no statistically significant difference between the groups in terms of the presence of significant coronary artery disease (*p* = 0.179). In addition, the patients were evaluated regarding the total number of affected vessels in terms of coronary artery disease, and no significant difference was detected between the groups in terms of the total number of affected vessels (*p* = 0.312) (Table 4).

Atherosclerotic involvement was evaluated for four major vessels (RCA, LMCA, LAD, and LCX). There were anomalies in 157 (28.9%) of the 544 vessels, while there was no anomaly in 387 (71.1%). Atherosclerotic involvement was observed in 69 vessels without anomalies (17.8%) and 45 vessels with anomalies (28.7%). The rate of atherosclerotic involvement in vessels with anomalies was significantly higher when normal and anomalous vessels were compared in terms of atherosclerotic involvement (*p* = 0.005). Furthermore, vessels were also compared in terms of having 50% or more stenosis. The rate of stenosis of 50% or more in vessels with anomalies was significantly higher than that in normal vessels (*p* = 0.023). The findings are presented in Table 5.

## 4. Discussion

CAAs are extremely rare in the general population. Coronary abnormalities do not have a standard nomenclature or categorization system. In our study, the categorization system described by Greenberg et al. was used [10]. There are also publications in the literature that do not accept myocardial bridging and coronary ectasia as coronary anomalies, instead considering them coronary anatomy variants [14,15]. The incidence of CAAs varies between 0.6% and 5.64%, depending on the population, definition, and type of imaging modality [16,17]. While the incidence was 1.30% in the largest angiographic study, which was conducted by Yamanaka and Hobbs, the rate was 5.64% in the prospective research by Angelini et al. [16,18]. In our study, the overall incidence of CAAs was 2.61%, and course anomalies were the most common type with an incidence of 1.86%. This was followed by origin anomalies with an incidence of 0.72% and termination anomalies with an incidence of 0.03%. The most common anomaly among the course anomalies was myocardial bridging with an incidence of 1.09%. This rate was close to the 0.9% incidence found in the study by Greenspan et al. [19]. It has been reported that typical or atypical angina can be seen in myocardial bridging and there is a high risk of myocardial infarction [3].

In our study, the incidence of arterial duplications, which was the second most common type, was 0.77%, and all of them were LAD duplications. This rate was very close to the 0.68% value found by Sidhu et al. [2]. Spindola-Franco et al. reported the incidence of LAD duplication as 1% and described four types in their conventional coronary angiography study [20].

The most common anomaly originated from the opposite sinus or noncoronary sinus of the coronary artery with an incidence of 0.37% in 19 patients among the origin anomalies. Among these anomalies, 14 patients had RCAs originating from the left coronary sinus with an incidence of 0.27%, and all had an inter-arterial course after termination. In previous studies, this rate ranged between 0.32% and 0.46% [2,21,22]. Angelini et al. found this rate to be 0.92% [18]. A potentially dangerous coronary abnormality is RCA’s aberrant origin in the left sinus. It has been reported that myocardial ischemia, syncope, ventricular tachycardia, myocardial infarction, and sudden death may occur at rest or during exercise as a result of intimal deterioration and vasospasm due to compression of the proximal RCA by the great arteries because of the inter-arterial course [16]. In two cases, LCX originating from the right sinus was seen with an incidence of 0.04%, and it demonstrated a retro-aortic course after termination. The incidence in our study was lower than that in previous studies [2,16,23]. This anomaly alone does not cause a functional deterioration in the myocardium, but care should be taken, especially in patients with obstructive coronary artery disease or aortic valve replacement [24]. Two patients had RCA originating from the noncoronary sinus with an incidence of 0.04%. This anomaly is rarely seen [25]. An LMCA originating from the right coronary sinus was observed in only one case and its incidence was 0.02%. Angelini et al. reported this rate to be 0.15% [18].

In one patient, the RCA emerged from the pulmonary trunk with an incidence of 0.02% (ARCAPA syndrome). An uncommon but serious congenital cardiac disorder is the origin abnormality of the RCA from the pulmonary artery. Cases are mostly asymptomatic and diagnosed at adult ages [26]. Two patients had a single coronary artery anomaly with an incidence of 0.04% and it originated from the right coronary sinus. It is unusual for a single coronary artery to originate from the right coronary sinus. Similar cases were described by Özyurtlu et al. and Pergola et al., and studies show that they comprised 3.3% of all coronary anomalies [27,28].

Although it was reported as the most common coronary artery anomaly in several previous studies, multiple ostium anomalies in which the LMCA was not observed and the LAD and LCX originate with ostia separate from the left coronary sinus were the fourth most frequently detected coronary artery anomaly in our study, with 12 patients and an incidence of 0.23%. This rate was lower than those reported in previous studies [2,16,17]. Four patients had RCA anomalies with a high incidence of 0.08%, and their incidence was lower than those in previous studies [2,16,23].

Coronary artery fistulas are the termination of one or more of the usually enlarged and tortuous coronary arteries in a low-pressure vascular bed, such as the heart chambers, vena cava, coronary sinus, pulmonary artery, and bronchial vessels, and they are rare in adults [2,29]. The incidence of coronary artery fistula has been reported to vary between 0.05% and 0.9%. RCA fistulas are the most common, and right-sided cardiac cavities and vessels are the most common termination sites [30]. Fistulas are usually small, do not cause significant hemodynamic effects, and are detected incidentally during cardiac catheterization or echocardiography. If the fistulas are large, they may cause shunts and cause fistula rupture, coronary steal, heart failure, or pulmonary hypertension [2]. In our study, fistula incidence was seen in two cases, 0.03%. This incidence, which was the same as that found by Ganga et al. [1], was similar to that seen in many studies [11,16,22]. In one case, there was a fistula and accompanying PDA aneurysm between the left atrium and the PDA. In the other case, there was a fistula between the LAD and the pulmonary artery. The fistulas in our study were small and did not cause a significant shunt. We did not have any patients with noncardiac termination anomalies.

There are studies in the literature investigating atherosclerotic involvement in normal and abnormal coronary arteries using catheter angiography, and incidence has been reported between 1.7% and 72.2% [2,11,12,21,23,24]. In addition to the importance of MDCTCA and catheter angiography in the diagnosis of CAA, cardiac magnetic resonance imaging (MRI) with virtual angioscopy is an important tool for demonstrating abnormal coronary anatomy and myocardial function and evaluating late gadolinium uptake and ischemia-induced fibrosis. There are also studies showing that it can be used in the initial and postoperative evaluation of children with CAAs [31].

In patients with coronary anomalies, nonpharmacological functional imaging (e.g., nuclear study, echocardiography, or stress cardiac MRI) is recommended to confirm/exclude myocardial ischemia [32]. Moreover, it has been shown that normal stress test results obtained using only stress ECG instead of sensitive methods such as nuclear perfusion imaging or stress echocardiography do not prevent sudden cardiac death. It has been shown that patients with CAAs but no coronary artery disease who underwent surgery had ischemia before surgical repair. This suggests that there is a relationship between coronary anomalies and ischemia, and ischemia can be detected by perfusion imaging [33]. However, no standard protocol has been proposed thus far to classify CAA-associated ischemia due to the limited number of studies.

We conducted our study using the MDCTCA and CAD-RADS scoring system, contrary to previous studies using catheter angiography. We investigated whether there was a difference between the anomaly groups in terms of CAD-RADS scores. Since there were two patients with termination anomalies, we compared atherosclerotic involvement only in patients with origin and course anomalies. There was no significant difference between the groups in terms of atherosclerotic involvement. We have not encountered any study in the literature that makes such a comparison. Considering that MDCTCA is being used with increasing frequency in recent years, we think that our study will be a guide for similar studies to be conducted in the future.

We found both atherosclerotic involvement and 50% or more involvement higher in vessels with anomalies compared to normal vessels, while no difference was detected between the anomalies. There are studies in the literature demonstrating that atherosclerotic involvement is high in vessels with anomalies, similar to our study [2,11,12].

MDCTCA is helpful both in the diagnosis of CAAs and in the early detection and development of prevention strategies for coronary atherosclerosis. We think that prospective studies with larger series are needed to investigate CAAs with MDCTCA and atherosclerotic involvement in individuals with CAAs.

## 5. Limitations

The data we obtained are limited and may not represent the general population since our study was retrospective and based on a single center.

## Figures and Tables

**Table 1:** Classification of congenital coronary artery anomalies by Greenberg et al. [10].

Congenital Coronary Artery Anomalies
**A.** **Origin Anomalies**
1. High take-off
2. Multiple ostium
3. Single coronary artery
4. Orgination of the coronary artery from the pulmonary artery
5. Orgination of the coronary artery from the contralateral or non-coronary sinus
**B.** **Course Anomalies**
1. Myocardial bridging
2. Artery duplication
**C.** **Termination Anomalies**
1. Coronary arterial fistulas
2. Non-cardiac termination

**Table 2:** Types and percentage distributions of coronary artery anomalies of the patients.

Coronary Artery Anomalies	n (%)
**Origin Anomalies n = 37 (27.2%)**	
**High take-off**	**3 (2.2)**
High take-off RCA	3(2.2)
**Multiple ostium**	**12 (8.8)**
No LMCA + LAD and LCX orginating from left coronary sinus	12 (8.8)
**Single coronary artery**	**2 (1.5)**
**Origin of the coronary artery from the pulmonary artery**	**1 (0.7)**
RCA orginating from the pulmonary artery (ARCAPA syndrome)	1(0.7)
**Origin of the coronary artery from the contralateral or non-coronary sinus**	**19 (13.9)**
RCA orginating from the left coronary sinus	14 (10.2)
LCX orginating from the right coronary sinus	2 (1.5)
LMCA orginating from the right coronary sinus	1 (0.7)
RCA orginating from the non-coronary sinus	2 (1.5)
**Course Anomalies n = 97 (71.3%)**	
**Myocardial bridging**	**57 (41.9)**
LAD myocardial bridging	54 (39.7)
LAD and LCX myocardial bridging	3 (2.2)
**Artery duplication**	**40 (29.4)**
LAD duplication	40 (29.4)
**Termination Anomalies n = 2 (1.5%)**	
**Coronary arterial fistulas**	**2 (1.5)**
Left atrium-PDA fistula	1 (0.75)
Pulmonary artery-LAD fistula	1 (0.75)
**Non-cardiac termination**	**0 (0)**
**Total n = 136 (100%)**	**136 (100)**

**Table 3:** Demographic characteristics and coronary risk factor status of patients with coronary artery anomalies.

Feature	Origin A	Course A	Termination A	Total	*p* *
	(n = 37)	(n = 97)	(n = 2)	(n = 136)	
	n (%)	n (%)	n (%)	n (%)	
Female	18 (48.6)	38 (39.2)	2 (100)	58 (42.6)	0.320
Male	38 (39.2)	59 (60.8)	0 (0)	78 (57.4)	
Mean Age ± SD	59.1 ± 11.9	57.0 ±12.0	60.5 ± 7.8	57.6 ± 11.9	0.373
Hypertension	21 (56.8)	43 (44.3)	0 (0)	64 (47.1)	0.198
Diabetes Mellitus	3 (8.1)	22 (22.7)	0 (0)	25 (18.4)	0.053
Hypercholesterolemia	20 (54.1)	39 (40.2)	1 (50)	60 (44.1)	0.149
Smoking history	10 (27.0)	38 (39.2)	0 (0)	48 (35.3)	0.190
Family history	18 (48.6)	40 (41.2)	0 (0)	58 (42.6)	0.439

n: Number of patients; A: Anomaly. * Termination anomalies are not included when calculating *p*-values.

**Table 4:** The patients’ CAD-RADS scores and the number of affected vessels in terms of coronary artery disease.

Feature	Origin A	Course A	Termination A	Total	*p* **
	(n = 37)	(n = 97)	(n = 2)	(n = 136)	
	n (%)	n (%)	n (%)	n (%)	
CAD-RADS 0	16 (43.2)	60 (61.9)	1 (50.0)	77 (56.6)	
CAD-RADS 1	8 (21.6)	13 (13.4)	1 (50.0)	22 (16.2)	
CAD-RADS 2	5 (13.5)	12 (12.4)	0 (0)	17 (12.5)	
CAD-RADS 3	4 (10.8)	9 (9.3)	0 (0)	13 (9.6)	0.220
CAD-RADS 4	3 (8.1)	3 (3.1)	0 (0)	6 (4.4)	
CAD-RADS 5	1 (2.7)	0 (0)	0 (0)	1 (0.7)	
CAD-RADS 0,1,2	29 (78.4)	85 (87.6)	2 (100)	116 (85.3)	0.179
CAD-RADS 3,4,5	8 (21.6)	12 (12.4)	0 (0)	20 (14.7)	
Number of vessels					
affected *					
1	10 (27.0)	15 (15.5)	1 (50)	26 (19.1)	
2	5 (13.5)	11 (11.3)	0 (0)	16 (11.8)	
3	5 (13.5)	6 (6.2)	0 (0)	11 (8.1)	0.312
4	1 (2.7)	4 (4.1)	0 (0)	5 (3.7)	
5	0 (0)	0 (0)	0 (0)	0 (0)	
6	0 (0)	1 (1.0)	0 (0)	1 (0.7)	

A: Anomaly; n: number of patients. *: RCA, LMCA, LAD, LCX, PDA, and PLD included. **: Termination anomalies are not included when calculating *p*-values.

**Table 5:** Comparison of normal and anomalous vessels in terms of atherosclerotic involvement.

Characteristic	Normal Vessel Total n = 387 n (%)	Vessel with Anomaly Total n = 157 n (%)	*p*
Atherosclerotic involvement	69 (17.8)	45 (28.7)	0.005
No atherosclerotic involvement	318 (82.2)	112 (71.3)	
≥50% stenosis	14 (3.6)	13 (8.3)	0.023
Normal or <50% stenosis	373 (96.4)	144 (91.7)	

## Data Availability

The data supporting this article are available from the corresponding author upon reasonable request.
